# Scheimpflug imaging of the anterior segment following simultaneous cross-linking with topography-guided custom ablation treatment for keratoconus

**DOI:** 10.5935/0004-2749.20220024

**Published:** 2025-08-21

**Authors:** Dorukcan Akincioglu, Gokhan Ozge, Gokcen Gokce, Onder Ayyildiz, Umut Karaca, Fatih Mehmet Mutlu

**Affiliations:** 1 Department of Ophthalmology,Ataturk State Hospital, Antalya, Turkey; 2 Department of Ophthalmology, Gulhane School of Medicine, University of Health Sciences, Ankara, Turkey; 3 Department of Ophthalmology, Memorial Hospital, Kayseri, Turkey; 4 Department of Ophthalmology, Gülhane Training and Research Hospital, Ankara, Turkey; 5 Department of Ophthalmology, Süleyman Demirel University, Isparta, Turkey

**Keywords:** Anterior, Keratoconus, Photorefractive, Phototherapeutic, Scheimpflug, Ceratocone, Segmento anterior do olho, Reagentes para ligações cruzadas, Acuidade visual

## Abstract

**Purpose:**

To report alterations in the anterior segment following accelerated corneal collagen cross-linking and topo-guided customized ablation treatment with the Nidek vision excimer laser system (Nidek Co., Ltd., Gamagori, Japan) in a single procedure.

**Methods:**

We reviewed the medical records of patients who underwent cross-linking for progressive keratoconus. We divided patients into four groups based on the treatment protocol. Eyes were evaluated regarding uncorrected distance visual acuity, corrected distance visual acuity, keratometry (maximum [K_max_], equivalent keratometry readings, K_steep_ and K_flat_ parameters), corneal elevations (anterior and posterior), the anterior radius of curvature, the posterior radius of curvature, anterior chamber volume, anterior chamber depth, anterior chamber angle and the pachymeter of the thinnest locale of the cornea before the surgery and at 1, 3, 6, and 12 months after the procedure.

**Results:**

We included 259 eyes of 227 patients with progressive keratoconus who underwent treatment. The mean respective baseline uncorrected distance visual acuity and corrected distance visual acuity were: 0.68 ± 0.45 and 0.34 ± 0.40 in Group 1; 0.82 ± 0.44 and 0.33 ± 0.23 in Group 2; 0.61 ± 0.36 and 0.21 ± 0.17 in Group 3; and 0.65 ± 0.38 and 0.23 ± 0.18 in Group 4; logMAR did not show significant difference among the groups (p=0.14 and p=0.06, respectively). Visual improvements were better in the combined surgery groups. Mean K_max_ in Groups 1, 2, 3, and 4 were 57.24 ± 7.51, 59.26 ± 6.94, 53.73 ± 4.60, and 54.31 ± 4.25 diopter (D), respectively. Group 1 demonstrated increased K_max_ for 6 months. Maximum flattening by 3.38 ± 2.35 D 1 year after surgery was observed in Group 4 (p<0.05). Decreased anterior chamber angle, anterior chamber depth, and anterior chamber volume were similar, indicating the stability of the anterior chamber.

**Conclusion:**

Visual and anatomical improvement is better, with improved stability of the anterior segment, in combined surgery groups compared with cross-linking alone.

## Abstract (pt)

**Objetivo:**

Relatar alterações no segmento anterior após *cross-linking* acelerado de colágeno da córnea e tratamento de ablação personalizado guiado por topografia com sistema laser de excimer de Nidek em um único procedimento.

**Métodos:**

Foram revisados os prontuários de pacientes submetidos ao *cross-linking* para ceratocone progressivo. Dividimos os pacientes em quatro grupos com base no protocolo de tratamento. Os olhos foram avaliados quanto à distância da acuidade visual não corrigida, distância da acuidade visual corrigida, ceratometria (K_max_, leituras de ceratometria equivalentes, parâmetros K_íngreme_ e K_plano_), elevações da córnea (anterior e posterior), raio anterior da curvatura, raio posterior da curvatura, volume da câmara anterior, profundidade da câmara anterior, ângulo da câmara anterior e paquímetro do ponto mais fino da córnea antes da cirurgia e com 1, 3, 6 e 12 meses após o procedimento.

**Resultados:**

Foram incluídos duzentos e cinquenta e nove olhos de 227 pacientes com ceratocone progressivo submetidos a tratamento. A média da distância da acuidade visual não corrigida e a média da distância da acuidade visual corrigida foram 0,68 ± 0,45 e 0,34 ± 0,40 no grupo-1, 0,82 ± 0,44 e 0,33 ± 0,23 no grupo-2, 0,61 ± 0,36 e 0,21 ± 0,17 no grupo-3, 0,65 ± 0,38 e 0,23 ± 0,18 no grupo-4 em LogMAR sem diferença significativa entre os grupos (p=0,14 e p=0,06). Melhoras visuais foram superiores em grupos de cirurgia combinada. A média de K_max_ em dioptria no grupo 1, grupo 2, grupo 3 e grupo 4 foi de 57,24 ± 7,51, 59,26 ± 6,94, 53,73 ± 4,60 e 54,31 ± 4,25 respectivamente. O grupo 1 demonstrou aumento do K_max_ por seis meses. Máximo achatamento foi observado no grupo 4 por 3,38 ± 2,35 D 1 ano após a cirurgia (p<0,05). A diminuição do ângulo da câmara anterior, da profundidade da câmara anterior e do volume da câmara anterior foi semelhante, indicando a estabilidade da câmara anterior.

**Conclusão:**

A melhora visual e anatômica é superior com a estabilidade melhorada do segmento anterior em grupos de cirurgia combinada em comparação com o *cross-linking* isolado.

## INTRODUCTION

The cornea contributes two-thirds of the refractive power of the eye, and a clear cornea with optimal curvature and symmetry is the fundamental component allowing light to enter the eye and form a real image on the light-sensing retina. Keratoconus (KC) is a degenerative, progressive disorder of the cornea characterized by thinning and irregular corneal topography^(1)^. Disruption of the corneal collagen network leads to degeneration with subsequent surface curvature irregularities and decreased vision. Due to abnormal corneal biomechanics, anterior segment parameters change and contribute to compromised vision with wavefront aberrations. Corneal collagen cross-linking (CXL) is a procedure applied to initiate the formation of new molecular bonds between the collagen fibrils, resulting in increased corneal biomechanical strength^(2)^. Conventional CXL and some modalities of CXL are useful in halting progression with significant reductions in topographic data^(3)^. Moreover, protocols (i.e., Athens protocol^(4)^, Cretan protocol^(5)^, and Cretan protocol plus^(6)^) combining CXL with selective excimer laser ablation are described. However, different excimer laser platforms have different ablation patterns and algorithms for topo-guided ablations.

In this retrospective study, we aimed to evaluate anterior segment parameters in patients with KC who underwent a combination of topo-guided customized ablation treatment and CXL in a single procedure using Scheimpflug imaging.

## METHODS

This was a retrospective study conducted in accordance with the tenets of the Declaration of Helsinki, with approval obtained from the institutional review board of Gülhane Training and Research Hospital (Ankara, Turkey). Written informed consent was provided by each patient or patients’ parents (for those aged <18 years). We reviewed the medical records of patients with progressive KC who underwent CXL surgery. In our study, documented progressive KC was defined as an increase in the topographic steepest keratometry value by >1 diopter (D) in the previous 6 months. The ABCD grading system, a newer tomographic method for staging KC, was used^(7)^. We recorded the uncorrected distance visual acuity (UDVA), corrected distance visual acuity (CDVA), keratometry (maximum [K_max_], equivalent keratometry readings, K_steep_ and K_flat_ parameters), anterior radius of curvature, posterior radius of curvature, anterior chamber volume (ACV), anterior chamber depth (ACD), anterior chamber angle (ACA), pachymetry of the thinnest locale of the cornea, and corneal elevations (anterior and posterior) prior to the surgery and at 1, 3, 6, and 12 months after the procedure.

The four groups based on treatment protocols were: Group 1, CXL; Group 2, CXL+phototherapeutic keratectomy (PTK); Group 3, CXL+photorefractive keratectomy (PRK); and Group 4, CXL+PTK+PRK.

### Inclusion and exclusion criteria

The inclusion criteria were: progressive KC in patients who underwent surgery and follow-up for ≥1 year; and baseline corneal thickness of >400 µm. The exclusion criteria were: history of previous eye surgery; any other ophthalmic pathology; and corneal scarring.

### Examination and measurements

The preoperative examination included UDVA, CDVA, manifest and cycloplegic refractions, slit-lamp evaluation, tonometry, and fundoscopy examination. We used both Oculus Pentacam^®^ topography (Oculus Optikgerate GmbH, Wetzlar, Germany), which is a noninvasive device determining the topography and pachymetry of the entire cornea using a 360° rotating Scheimpflug camera and Optical Path Difference (OPD-Scan III^®^; Nidek Co., Ltd., Gamagori, Japan) scanning system. We stopped the use of soft and rigid contact lenses 2 and ≥4 weeks prior to examination/surgery, respectively. For treatment, we used a custom ablation transition zone (CATz) ablation profile (Quest; Nidek^®^ Co., Ltd.) and the final fit software (Nidek^®^ Co., Ltd.) based on maps obtained from the linked topography device OPD-Scan III^®^. We measured both anterior and posterior corneal elevations by using an 8.0-mm diameter area to calculate the best-fit sphere derived, enhanced reference surfaces fixed to the corneal apex, as displayed by Bellin-Ambrossio Enhanced Ectasia Display (BAD) available on the Pentacam (OCULUS^®^ GmbH, Wetzlar Germany).

### Surgical technique

After the administration of topical anesthesia with proxymetacaine hydrochloride 0.5% eye drops (Alcaine; Alcon, Inc., Hünenberg, Switzerland), we performed transepithelial PTK, PRK or PTK following PRK using the Nidek Vision excimer laser system (Nidek^®^ Co., Ltd.). The diameter of the effective optical zone decreased to 5.5 mm, and the transition zone was 1.5 mm. We mechanically removed central 8 mm of the corneal epithelium and performed corneal pachymetry using an ultrasound pachymeter. We instilled a riboflavin (RF) solution with hydroxypropyl methylcellulose in the center of the cornea for 30 min (one drop every 2 min); of note, this was a dextran-free solution (0.1% riboflavin + hydroxypropyl methylcellulose, Mediocross M^®^; Avedro Inc., Waltham, MA, USA). At the end of the instillation period, the cornea was exposed to UVA/365 nm light at an incident intensity of 18 mW/cm^2^ for 5 min with a total energy dose of 5.4 J/cm^2^. We placed a bandage soft contact lens on the cornea at the end of UVA exposure, which was removed after complete reepithelialization.

Postoperatively, we administered topical antibiotic moxifloxacin (Vigamox^®^; Alcon Inc.) four times daily for the first week. This was followed by administration of topical nonsteroidal anti-inflammatory drops Nepafenac 0.1% (Nevanac^®^; Alcon Research Ltd., Fort Worth, TX, USA) four times daily for the first month, and artificial tears with no preservation (Refresh^®^; Allergan Inc., Irvine, CA, USA) six times daily for the first month and as needed. Following complete epithelial healing, we added loteprednol etabonate 0.5% (Lotemax^®^; Bausch+Lomb) drops four times daily to the treatment regimen for4 weeks.

We used the SPSS version 20.0 for Windows software (IBM Corp., Armonk, NY, USA) for statistical analysis. The Shapiro-Wilk test was employed to test the normality of the parameters, the paired sample t-test was used to compare preoperative and postoperative variables in each group, and one-way analysis of variance was utilized to compare groups. We calculated the relative odds of favorable surgical outcome for a variable of interest using logistic regression analysis. The Pearson or Spearman’s test was used for correlation analysis related to the distribution of the parameters. The Hosmer-Lemeshow goodness-of-fit test was used to assess the quality of the logistic regression model.

## RESULTS

**Figure 1:**
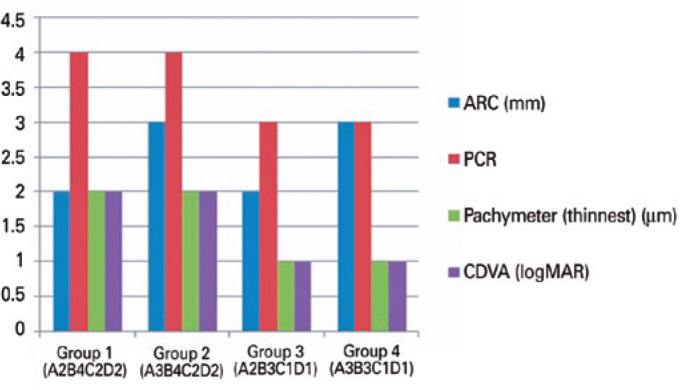
Keratoconus grading of patients based on the ABCD system described by Bellin et al.^(7)^.

**Figure 2:**
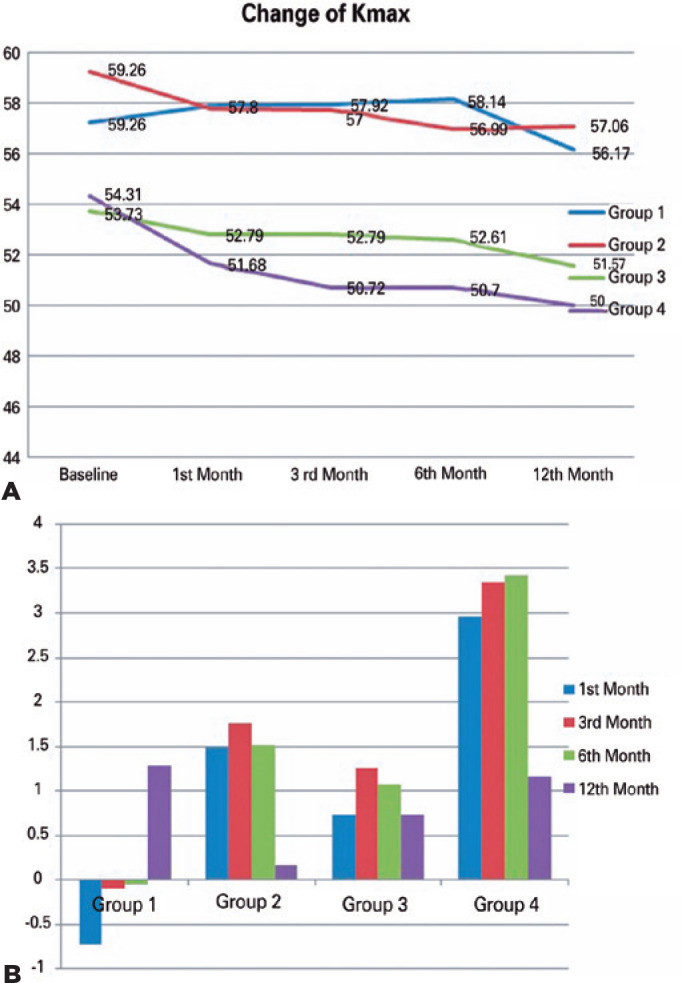
Baseline and postoperative maximum keratometric (K_max_) readings. (A) Mean preoperative and postoperative interval maximum keratometric readings considering four surgical planning strategies. (B) Maximum keratometry difference between preoperative and postoperative intervals (K_max_ preoperative - K_max_ postoperative).

**Figure 3:**
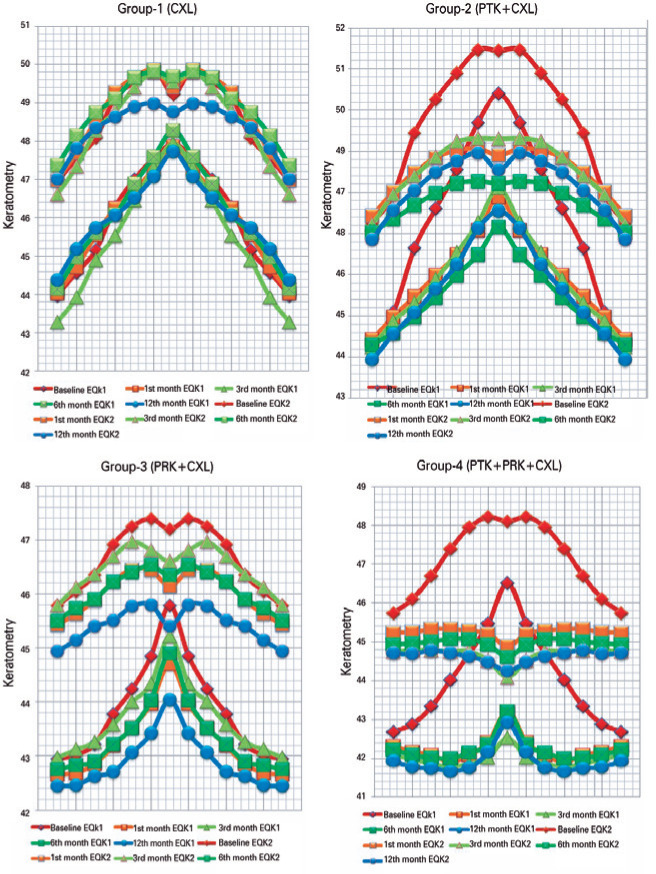
Corneal equivalent keratometry (EqK) readings in 1/2/3/4/5/6/7 mm. Keratometry readings are shown as corneal surface. Central represents 1-mm EqK and both sides represent 2-mm EqK, and subsequently 3, 4, 5, 6, and 7 mm. Steep and flat keratometry are presented in the same color in the relevant month. A major change is observed in the combined surgical groups. Red color represents baseline keratometric readings. The maximum change in keratometric readings and corneal morphology was observed in Group 4.

**Figure 4:**
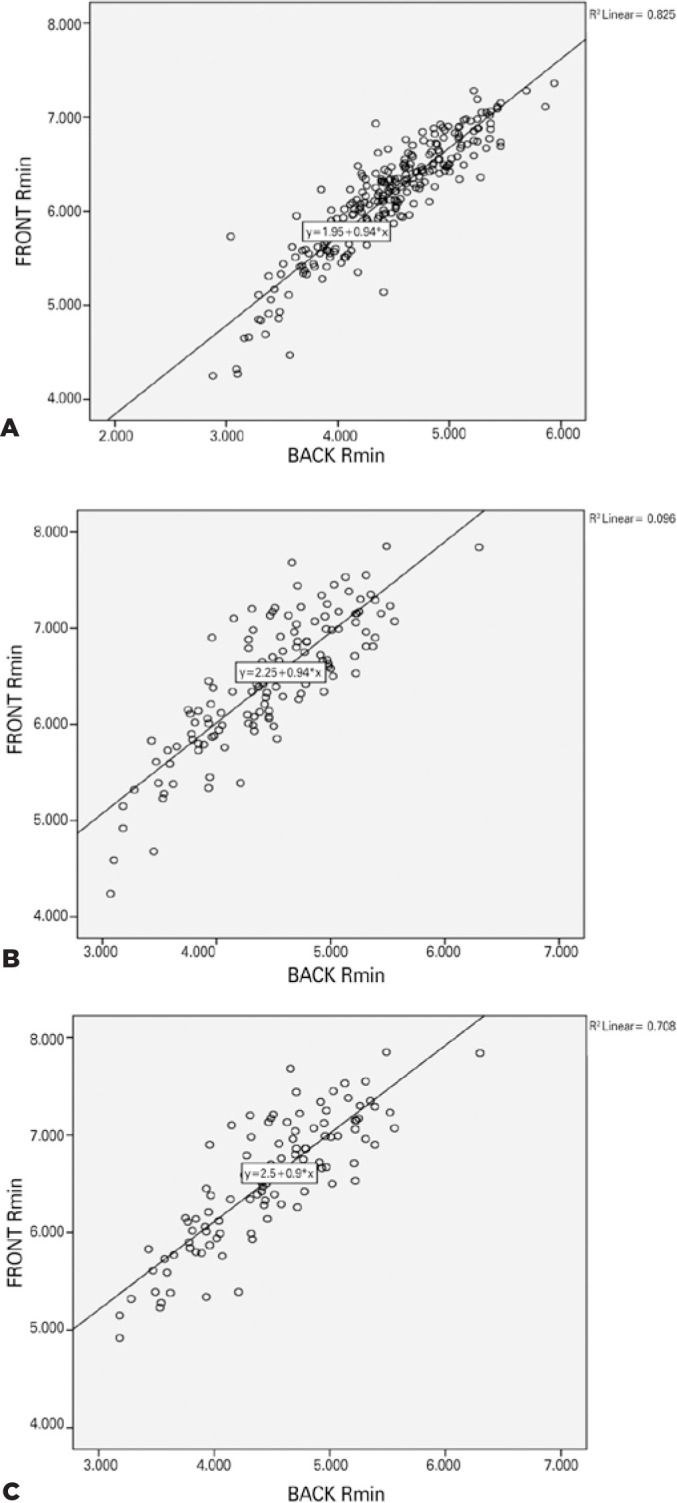
(A) Baseline correlation between the minimum curvature radius (R_min_) of the front and posterior surfaces. (B) Correlation between the minimum curvature radius of the front and posterior surfaces at 12 months. (C) Correlation between the minimum curvature radius of the front and posterior surfaces at 12 months, after excluding Group 1 (CXL).

This study included 259 eyes of 227 patients. Table 1 presents the baseline demographics and corneal morphology. Figure 1 presents all patients based on the ABCD grading system described by Belin et al.^(77)^. Respective postoperative UDVA and CDVA were: 0.51 ± 0.38 and 0.27 ± 0.32 in Group 1; 0.42 ± 0.47 and 0.10 ± 0.14 in Group 2; 0.24 ± 0.27 and 0.09 ± 0.13 in Group 3; and 0.24 ± 0.22 and 0.08 ± 0.11 in Group 4. Visual improvements were significantly different among groups due to the poor visual outcome in Group 1 (p=0.005). We observed the most significant visual improvements in Group 2. However, the differences in visual improvements in patients who underwent topo-guided customized ablation treatment and CXL were not statistically significant between each other (p=0.97). The respective mean UDVA and CDVA improvements were: 0.10 ± 0.22 and 0.08 ± 0.21 in Group 1; 0.36 ± 0.34 and 0.24 ± 0.13 in Group 2; 0.30 ± 0.27 and 0.10 ± 0.11 in Group 3; and 0.35 ± 0e.28 and 0.12 ± 0.13 in Group 4. Comparison of preoperative and postoperative K_max_ values showed a statistically significant decrease during 12 months in Groups 2, 3, and 4 (p<0.05). In contrast, the K_max_ value increased for 6 months in Group 1. Intergroup analysis showed a statistically significant difference between the groups (p<0.05). Post-hoc analysis revealed that this difference was related to a more significant decrease in Group 4 in the 12th month (Group 1: 0.31 ± 1.46 D; Group 2: 1.31 ± 3.52 D; Group 3: 1.20 ± 2.31 D; and Group 4: 3.38 ± 2.35 D) (Figure 2). Figure 3 presents preoperative and postoperative equivalent keratometry readings in a 1-/2-/3-/4-/5-/6-/7-mm zone. The mean preoperative back radius of minimum curvature (R_min_) was 4.32 ± 0.60 mm (Group 1), 4.09 ± 0.65 mm (Group 2), 4.57 ± 0.51 mm (Group 3), and 4.54 ± 0.48 mm (Group 4). A nonsignificant decrease of the back R_min_ was observed, followed by a nonsignificant increase in the 12th month. Front R_min_ and back R_min_ were highly correlated both preoperatively and postoperatively (Figure 4).

**Table 1:** Baseline demographics and corneal morphology

Group 1		Group 2	Group 3	Group 4	p-value
Sex	39.20%	27.80%	18.70%	19.60%	>0.05
Female	60.80%	72.20%	81.30%	80.40%	
Male					
Fellow eye					
Forme fruste KC	4%	5.60%	5.30%	1%	
Subclinical KC	11.80%	11.20%	17.30%	14.40%	
Clinical KC	80.20%	74.80%	76.10%	84.60%	
Donor cornea	4%	8.40%	1.30%	N/A	
Age (mean ± SD)	24.86 ± 6.09	29.47 ± 10.14	26.20 ± 7.76	27.83 ± 8.47	>0.05
UDVA (logMAR)	0.68 ± 0.45	0.82 ± 0.44	0.61 ± 0.36	0.65 ± 0.38	>0.05
CDVA (logMAR)	0.34 ± 0.40	0.33 ± 0.23	0.21 ± 0.17	0.23 ± 0.18	>0.05
ARC (mm)	6.52 ± 0.65	6.25 ± 0.54	6.83 ± 0.41	6.77 ± 0.43	<0.001
PRC (mm)	4.88 ± 0.56	4.66 ± 0.54	5.13 ± 0.37	5.07 ± 0.43	<0.001
Pachymeter (thinnest) (mm)	425.64 ± 76.35	436.36 ± 35.05	456.30 ± 36.01	465.89 ± 29.71	<0.001
Pachymeter (highest point) (mm)	483.23 ± 63.13	442.19 ± 40.30	482.81 ± 56.86	476.85 ± 61.12	<0.001
Front K1 (D)	47.70 ± 5.71	49.44 ± 4.36	44.91 ± 2.50	45.22 ± 2.56	<0.001
Front K2 (D)	50.97 ± 5.80	52.45 ± 4.68	48.74 ± 3.10	49.29 ± 2.86	<0.001
Anterior chamber volume (mm^3^)	196.88 ± 43	189.83 ± 29.65	208.68 ± 35	199.88 ± 36.24	>0.05
Anterior chamber depth (mm)	3.37 ± 0.42	3.23 ± 0.58	3.34 ± 0.47	3.33 ± 0.31	>0.05
Anterior chamber angle (°)	37.64 ± 6.61	38 ± 6.27	39.30 ± 5.01	38.84 ± 6.03	>0.05
Front K_max_ (D)	57.24 ± 7.51	59.26 ± 6.94	53.73 ± 4.60	54.31 ± 4.25	<0.001
Back R_min_ (mm)	4.32 ± 0.60	4.09 ± 0.65	4.57 ± 0.51	4.54 ± 0.48	<0.001
Ablation depth (mm)	N/A	58.29 ± 15.13 (PTK)	30.35 ± 11.68 (PRK)	46.42 ± 17.29/26.39 ± 9.02 (PTK/PRK)	
Forward elevation (mm)	23.98 ± 13.23	27.97 ± 10.88	18.98 ± 8.25	20.89 ± 8.85	<0.001
Backward elevation (mm)	54.58 ± 23.22	62.33 ± 20.64	44.17 ± 16.18	47.32 ± 17.06	<0.001

ARC= anterior radius of curvature; CDVA= corrected distance visual acuity; D= diopter; K1= flat keratometry; K2= steep keratometry; KC= keratoconus; K_max_= maximum keratometry; N/A= not applicable; PRC= posterior radius of curvature; PRK= photorefractive keratectomy; PTK= phototherapeutic keratectomy; R_min_= radius of minimum curvature; SD= standard deviation; UDVA= uncorrected distance visual acuity.

ARC= anterior radius of curvature; CDVA= corrected distance visual acuity; PRC= posterior radius of curvature.

CXL= collagen cross-linking; PRK= photorefractive keratectomy; PTK= phototherapeutic keratectomy.

CXL= collagen cross-linking.

**Figure 5:**
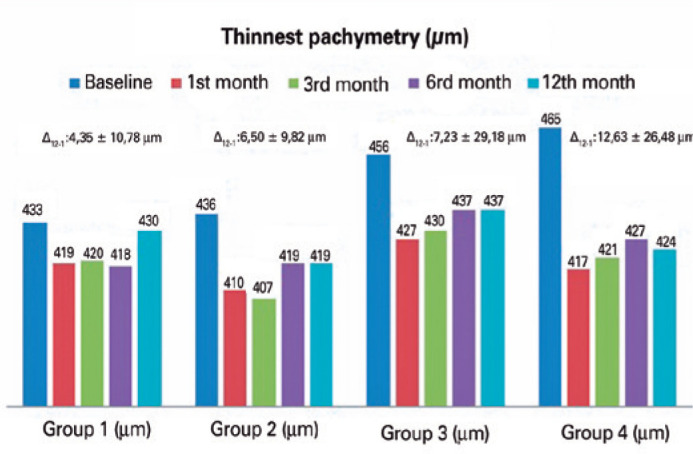
Decreased pachymetry of the corneal thinnest location was observed in all groups. Pachymetry at 1 month was thinner compared with baseline; however, the thickness increased thereafter.

The mean preoperative ACV was 200.44 ± 36.82 mm^3^. The mean postoperative ACV values at 1, 3, 6, and 12 months were 197.46 ± 38.13 mm^3^, 193.99 ± 38.13 mm^3^, 195.66 ± 38.70 mm^3^, and 196.25 ± 38.46 mm^3^, respectively. The decrease of ACV was statistically significant and similar between the groups. The ACA decreased significantly in Group 4 (p<0.001). Nevertheless, at 12 months, ACA measurements were similar between the groups (p>0.05). Preoperatively, the mean ACD was 3.33 ± 0.42 mm. Following surgery, the mean ACD values at 1, 3, 6, and 12 months were 3.34 ± 0.32 mm, 3.29 ± 0.39 mm, 3.33 ± 0.34 mm, and 3.28 ± 0.46 mm, respectively. Scheimpflug measurements did not show significant difference between the groups (p>0.05). At baseline, the pachymetry of the thinnest locale of the cornea was significantly different. Inevitably, postoperative pachymetry revealed significantly thinner corneas compared with preoperative pachymetry in all groups (p<0.001). Protocol comparison showed a statistically significant difference between the groups. Figure 5 presents pachymetry and postoperative changes. An increase in pachymetry measurements at 12 months compared with the first postoperative month was observed in all groups. The increases were 4.35 ± 10.78 µm, 6.50 ± 9.82 µm, 7.23 ± 29.18 µm, and 12.63 ± 26.48 µm in Groups 1, 2, 3, and 4, respectively (p=0.53). Anterior elevations decreased in all groups. Table 2 presents preoperative and postoperative elevations. All groups, except Group 1, demonstrated statistically significant decreases in corneal elevations following surgery (p<0.05). However, posterior corneal elevations had increased following surgery in Group 4 (p<0.001). A comparison of favorable surgical outcomes with preoperative anterior segment parameters is presented in table 3. Unfavorable surgical outcomes were 29.4%, 16.7%, 12%, and 5.2% in Groups 1, 2, 3, and 4, respectively. Logistic regression analysis demonstrated that none of the study variables in the equation had a significant coefficient for the formula Nagelkerke R_Square_: 0.46; χ^2^: 4.611; p=0.79.

**Table 2:** Baseline and postoperative interval corneal elevations (anterior and posterior)

	Group 1		Group 2		Group 3		Group 4		FE diff	BE diff
	FE max (pm)	BE max (µm)	FE max (µm)	BE max (pm)	FE max (µm)	BE max (pm)	FE max (µm)	BE max (pm)		
Baseline	28.31 ± 12.55	62.41 ± 22.33	32.69 ± 9.78	86.38 ± 108.66	26.52 ± 13.90	55.49 ± 22.55	26.60 ±11.33	56.22 ± 17.06		
1 month	26.77 ± 14.05	62.67 ± 22.42	30.39 ± 10.13	70.72 ± 21.11	22.97 ± 13.84	58.72 ± 20.99	21.28 ± 13.72	64.24 ± 18.27	p>0.05^**^	p 0.05^**^
3 months	30.43 ± 13.72	58.59 ± 19.16	30.15 ± 9.97	70.78 ± 20.28	23.43 ± 14.38	59.55 ± 21.89	26.10 ± 48.09	61.34 ± 19.02	p >0.05^**^	p<0.05^**^
6 months	29.02 ± 14.75	61.86 ± 21.62	30.42 ± 9.99	71.28 ± 20.12	25.25 ± 15.98	59.90 ± 28.36	22.04 ± 13.35	61.28 ± 16.06	P>0.05^**^	p<0.05^**^
12 months	27.11 ± 13.16	59.88 ± 24.49	30.17 ± 9.14	72.17 ± 18.53	21.35 ± 10.67	53.62 ± 18.04	22.00 ± 14.00	64.80 ± 17.74	p>0.05^**^	p<0.05^**^
	p>0.05^*^	p>0.05^*^	p<0.05^*^	p<0.05^*^	p<0.05^*^	p>0.05^*^	p<0.05^*^	p<0.05^*^		

* = comparison with baseline in each parameter. A paired-test was used;

** = comparison between groups. BE= back elevation; FE= front elevation; max= maximum.

**Table 3:** Surgical success following KC treatment

Parameter	Limit	p-value	OR (95% CI)
K max	<55 D	<0.001	4.63 (1.911-11.240)
THP	<450 pm	<0.05	0.35 (0.16-0.75)
ARC	<6.35 mm	<0.001	0.28 (0.13-0.60)
PRC	<5.15 mm	<0.001	0.16(0.05-0.49
ACV	<170 mm^3^	>0.05	1.15 (0.44-2.98)
ACD	<3 mm	>0.05	1.69 (0.48-5.89)
FE	<30 pm	<0.05	2.96(1.35-6.46)
BE	<60 pm	<0.001	5.31 (2.31-12.21)

ACD= anterior chamber depth; ACV= anterior chamber volume; ARC= anterior radius of curvature; BE= back elevation; CI= confidence interval; D= diopter; FE= front elevation; KC= keratoconus; K_max_= maximum keratometry; OR= odds ratio; THP= thinnest pachymeter; PRC= posterior radius of curvature.

∆_12-1_: change in pachymetry at 12 months and 1 month.

## DISCUSSION

KC is a progressive degenerative corneal disorder, generating a high degree of myopia and irregular astigmatism due to impaired corneal biomechanics and morphology. CXL is a treatment modality used to halt the progression of the disease. Nevertheless, patients continue to require effective visual rehabilitation following CXL. Photorefractive corneal surgeries and intraocular surgeries are performed for a better visual outcome^(8)^. In this study, we demonstrated and compared anterior segment parameters preoperatively and postoperatively following different treatment protocols.

Regarding K_max_, all groups showed improvement, which was more prominent in the combined surgery groups. We observed flattening mostly in Group 4 (PTK+PRK+CXL), followed by Group 2 (PTK+CXL) and Group 3 (PRK+CXL). The PTK procedure typically involves greater ablation depth compared with PRK. The ablation depth was 58.29 ± 15.13 µm, 30.35 ± 11.68 µm, and 46.42 ± 17.29 µm 26.39 ± 9.02 µm in Groups 2, 3, and 4, respectively. Hence, we hypothesized that the flattening effect was related to the extent of ablation, and this is more prominent in eyes that underwent PTK. Furthermore, visual improvements were better in patients who underwent topo-guided customized PTK. In this procedure, the cornea is reshaped with precise ablation. In Group 1, we observed an increasing K_max_ postoperatively. Kontadakis et al.^(9)^ described a similar postoperative change in the CXL Group, and this was attributed to postoperative epithelial hyperplasia with edema following the procedure. If this was related to edema following the procedure, we would expect an increase in pachymetry values. In our study, we recorded decreased pachymetry values in the first month and follow-ups for 12 months. We think that the increased K_max_ was related to irregular keratocyte activity during the first 6 months following CXL, which misguiding the scheimpflug camera.

Correlation between the anterior and posterior minimum radius of curvature was statistically significant at 12 months (r=0.83, p<0.001). Moreover, the change in the radius of curvature following surgery was statistically significant (r=0.33, p<0.001). However, the evaluation of groups regarding the correlation of changes in the anterior and posterior radius of curvature in Group 1 was not statistically significant (r=-0.03, p=0.86). Thus, anterior and posterior corneal asymmetry may increase following CXL, although the biomechanical transformation of the whole cornea is significant. Asymmetrical changes may cause unsatisfying visual improvement due to increased aberrations.

ACV, ACD, and ACA are parameters that may be indirectly affected by corneal biomechanical changes and a shifting iris lens diaphragm. ACD is statistically significantly increased in patients with KC^(10)^. Although studies have shown that ACV and ACA are also increased, the differences were not statistically significant^(11)^. Nonetheless, some experts claim that we may observe decrease in ACA due to compensating flattening of the peripheral cornea^(12)^. According to our literature review, an increased ACD is characteristic, mostly at the center and apex location. Steepening and flattening counterbalance one another; hence, the ACV and ACA may change depending on the localization of the cone and its radius of curvature. We recorded decreased ACV, ACD, and ACA in all groups, although this change was not statistically significant among groups and mostly similar after 6 months. These findings indicated stability of the anterior chamber parameters following different protocols of KC treatment.

Posterior corneal elevation with the Pentacam rotating scheimpflug camera is a novel marker for the diagnosis of KC^(13)^. These images are highly reproducible and repeatable^(14)^. In the present study, anterior corneal elevations decreased in all groups, and decreasing anterior corneal elevations were significantly correlated with flattening K_max_ (r=0.64, p<0.001). However, the correlation between posterior elevation and K_max_ was very slight and negative (r=-0.24, p<0.001). Posterior corneal elevations decreased in all groups except Group 4. In this group, posterior corneal elevations increased similarly to those noted in a study performed by Steinberg et al.^(15)^. Progressive increase of posterior corneal elevation was unexpected, although stabilization of the anterior part of the cornea was successful. The increasing posterior corneal elevations may be related to ongoing ectatic changes within the deeper layers of the cornea. We observed this unexpected change only in Group 4. Therefore, we hypothesized that this may be related to the extent of ablation, which was the greatest in Group 4. The correlation between the area of the ablated cornea and posterior corneal elevation was not statistically significant (p=0.16). However, frequency analysis showed that 61.4% of all eyes had increased posterior elevation following surgery. The rates were: 35.1% of the corneas in Group 1 with no ablation; 59.6% of the corneas with ablation depth <50 mm; and 72.6% of corneas with ablation depth ranging 50-100 mm. The correlation was nonsignificant, although the frequency increased with the area of the ablated cornea. We could not measure the biomechanical properties of the cornea, and this may be a limitation of our study.

In this study, patients who underwent CXL following PTK had significantly better visual improvements. Corneal parameters changed significantly among groups due to procedures; however, the anterior segment parameters (ACA, ACV, ACD) did not differ. Patients in Group 4 required a longer follow-up to observe any ongoing ectatic change because these corneas are already ectatic, and the safety limits of ablation are unknown. Furthermore, posterior elevation may not be a novel marker for postoperative follow-ups, contrary to preoperative follow-ups.
